# Final results of a phase I/II pilot study of capecitabine with or without vinorelbine after sequential dose-dense epirubicin and paclitaxel in high-risk early breast cancer

**DOI:** 10.1186/1471-2407-10-430

**Published:** 2010-08-16

**Authors:** Volkmar Müller, Christoph Thomssen, Marcus Schmidt, Manfred Glados, Christian Jackisch, Volker Heilmann, Axel Hinke, Antje Lehnert, Henryk Borowicz, Volker Möbus

**Affiliations:** 1 University Medical Center, Hamburg, Department of Gynecology, Martinistrasse 52, 20246 Hamburg, Germany; 2 University Clinic Halle (Saale), Department of Gynecology and Obestetrics, Magdeburger Str. 14, 06112 Halle (Saale), Germany; 3 Department of Obstetrics and Gynecology, University Medical Center of the Johannes Gutenberg-University, Medical School, Langenbeckstr. 1, 55131 Mainz, Germany; 4 Onkologische Schwerpunktpraxis, Südwall 20, 48653 Coesfeld, Germany; 5 Clinic for Gynecology and Obestetrics, Breast Center Offenbach, Starkenburgring 66, 63069 Offenbach, Germany; 6 University Clinic Ulm, Department of Obstetrics and Gynecology, Prittwitzstr. 43, 89075 Ulm, Germany; 7 WiSP Research Institute, Karl-Benz-Strasse 1, 40764 Langenfeld, Germany; 8 St. Josefs-Hospital, Department of Gynecology, Beethovenstrasse 20, 65189 Wiesbaden, Germany; 9 Staedtisches Klinikum Frankfurt-Hoechst, Gotenstraße 6-8, 65929 Frankfurt/M, Germany; 10 Written on behalf of the Arbeitsgemeinschaft Gynäkologische Onkologie (AGO) Breast Study Group

## Abstract

**Background:**

The integration of the non-cross-resistant chemotherapeutic agents capecitabine and vinorelbine into an intensified dose-dense sequential anthracycline- and taxane-containing regimen in high-risk early breast cancer (EBC) could improve efficacy, but this combination was not examined in this context so far.

**Methods:**

Patients with stage II/IIIA EBC (four or more positive lymph nodes) received post-operative intensified dose-dense sequential epirubicin (150 mg/m² every 2 weeks) and paclitaxel (225 mg/m² every 2 weeks) with filgrastim and darbepoetin alfa, followed by capecitabine alone (dose levels 1 and 3) or with vinorelbine (dose levels 2 and 4). Capecitabine was given on days 1-14 every 21 days at 1000 or 1250 mg/m^2 ^twice daily (dose levels 1/2 and 3/4, respectively). Vinorelbine 25 mg/m^2 ^was given on days 1 and 8 of each 21-day course (dose levels 2 and 4).

**Results:**

Fifty-one patients were treated. There was one dose-limiting toxicity (DLT) at dose level 1. At dose level 2 (capecitabine and vinorelbine), five of 10 patients experienced DLTs. Therefore evaluation of vinorelbine was abandoned and dose level 3 (capecitabine monotherapy) was expanded. Hand-foot syndrome and diarrhoea were dose limiting with capecitabine 1250 mg/m^2 ^twice daily. At 35.2 months' median follow-up, the estimated 3-year relapse-free and overall survival rates were 82% and 91%, respectively.

**Conclusions:**

Administration of capecitabine monotherapy after sequential dose-dense epirubicin and paclitaxel is feasible in node-positive EBC, while the combination of capecitabine and vinorelbine as used here caused more DLTs.

**Trial registration:**

Current Controlled Trials ISRCTN38983527.

## Background

Regimens containing both an anthracycline and a taxane are standard of care for patients with high-risk early breast cancer (EBC), such as those with four or more positive nodes [1]. Dose-dense administration appears to offer improved efficacy in node-positive disease [2,3]. In the Intergroup/CALGB 9741 trial, dose-dense doxorubicin, cyclophosphamide and paclitaxel significantly improved disease-free survival (DFS) compared with conventional dosing [2]. In a German Arbeitsgemeinschaft Gynäkologische Onkologie (AGO) trial, adjuvant dose-dense and dose-escalated sequential epirubicin (150 mg/m^2^), paclitaxel (225 mg/m^2^) and cyclophosphamide (2500 mg/m^2^) with granulocyte-colony stimulating factor support (dose-escalated sequential epirubicin, paclitaxel and cyclophosphamide [ddETC] regimen) improved DFS and overall survival compared with conventional therapy in patients with high-risk breast cancer. Although the ddETC regimen showed superior efficacy, the importance of the 2500 mg/m² cyclophosphamide dose remains unclear, since other studies failed to demonstrate a benefit of cyclophosphamide dose escalation when given at 3-weekly intervals. In addition, haematological toxicity in the ddETC regimen was most pronounced with cyclophosphamide [3]. Therefore, it is important to examine other compounds as partners for dose-dense anthracycline and taxane regimens, providing the rationale for this study.

Selection of appropriate chemotherapeutic agents in this context should be based on activity, tolerability and potential synergy. Capecitabine has demonstrated high activity in a range of treatment situations for metastatic breast cancer (MBC). Capecitabine monotherapy is effective after exposure to anthracyclines and taxanes [4], and significantly improves overall survival versus classical cyclophosphamide, methotrexate and 5-fluorouracil (CMF) as first-line therapy [5]. The addition of capecitabine to docetaxel in anthracycline-pretreated MBC improves overall survival [6]. However, in the adjuvant setting, doxorubicin-cyclophosphamide (AC) or CMF combination therapy were superior to capecitabine monotherapy [7]. Thus, although the integration of capecitabine into anthracycline and taxane combination regimens appears feasible, the optimal schedule of capecitabine-containing regimens in the adjuvant and neoadjuvant settings needs further investigation.

Another agent widely used in MBC is vinorelbine. Published data from trials evaluating vinorelbine-containing regimens suggest that replacing cyclophosphamide with vinorelbine as monotherapy may offer similar efficacy [8,9]. Although vinorelbine is often administered as monotherapy, several phase II studies in MBC have shown that the combination of vinorelbine and capecitabine is active and well tolerated [10-12]. The rationale for combining these two agents lies in their preclinical synergy [13], single-agent activity and differing safety profiles, with gastrointestinal effects and hand-foot syndrome predominating with capecitabine, and myelosuppression characterising vinorelbine therapy.

The present study was originally initiated as a feasibility study for the randomised, phase III trial to follow the ddETC study [3]. It was proposed that replacing high-dose cyclophosphamide with agents that are not cross-resistant to anthracyclines and taxanes might increase efficacy while avoiding cyclophosphamide-related toxicity.

## Methods

### Study objectives

The objective of the phase I part of the study was to assess the feasibility and tolerability of capecitabine with or without vinorelbine after intensified dose-dense sequential epirubicin and paclitaxel as adjuvant therapy in high-risk patients, and to identify the optimal dosing regimen. In the phase II part of the study, the primary objective was to determine 3-year DFS and 3-year overall survival. The study was conducted in accordance with the Declaration of Helsinki. The study was approved by the ethics committee State of Hessen, Germany (approval number 38/2003).

### Eligibility

Eligible patients were aged 18-65 years with histologically confirmed stage II/IIIA breast cancer with four or more positive axillary lymph nodes. All patients had undergone surgery (complete surgical resection [R0] of breast tumour and axilla) before inclusion in the study. Other key inclusion criteria included Eastern Cooperative Oncology Group (ECOG) performance status 0 or 1, left ventricular ejection fraction within the normal institutional range, and adequate haematological, renal and hepatic function. Patients were excluded if they had inflammatory breast cancer or had received neoadjuvant endocrine therapy, chemotherapy or radiotherapy. Patients with known dihydropyrimidine dehydrogenase deficiency or creatinine clearance < 30 mL/min were excluded. All patients provided written informed consent.

### Study treatment

Starting within 4 weeks after surgery, all patients received three cycles of epirubicin 150 mg/m^2^, day 1 every 14 days, with filgrastim 5 μg/kg on days 3-10, followed by three cycles of paclitaxel 225 mg/m^2^, given as a 3-hour infusion on day 1 every 14 days, with filgrastim as above. Patients then received four cycles of capecitabine days 1-14 either alone (dose levels 1 and 3) or in combination with vinorelbine at 25 mg/m² on days 1 and 8 of each 21-day cycle (dose levels 2 and 4). Dose levels were defined as follows: level 1: capecitabine 1000 mg/m²; level 2: capecitabine 1000 mg/m² plus vinorelbine 25 mg/m² days 1 and 8; level 3: capecitabine 1250 mg/m²; level 4: capecitabine 1250 mg/m² plus vinorelbine 25 mg/m² days 1 and 8.

The starting doses were selected because these or higher doses had demonstrated good tolerability in previous studies in the metastatic setting [11,12]. All patients received darbepoetin alfa from day 1 of chemotherapy until the end of radiotherapy (starting dose of 300 μg weekly for 4 weeks, after this every 3 weeks). Patients with hormone receptor-positive disease received tamoxifen 20 mg/day for 5 years after completion of chemotherapy.

**Figure 1:**
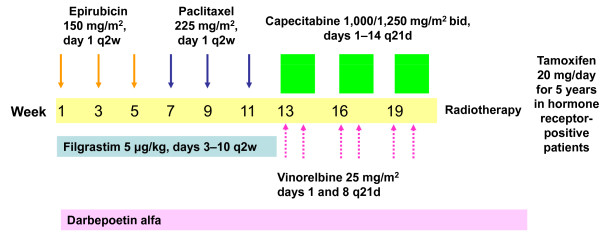
**Different parts of the treatment schedule**. Patients received dose-dense sequential epirubicin and paclitaxel, followed by capecitabine alone (dose levels 1 and 3) or with vinorelbine (dose levels 2 and 4).

Ten patients were to be enrolled to each dose level. If fewer than three of 10 patients experienced dose-limiting toxicity (DLT, defined as grade 4 febrile neutropenia, grade 4 diarrhoea or grade 3 diarrhoea lasting > 5 days, grade 3/4 neurotoxicity, grade 3 hand-foot syndrome, or treatment-related death), patients were enrolled to the next dose level. If three of 10 patients experienced a DLT, an additional five patients were enrolled to that dose level and if more than three experienced a DLT this level was to be rejected. The different parts of the treatment are outlined in Figure 1.

## Results

### Patient population

A total of 51 patients were enrolled in 10 centres between October 2003 and July 2006, all of whom were evaluable. Median age was 53 years (range 32-67) in the overall population, although patients in the lower two dose levels were typically slightly younger than those in the upper two dose levels (Table 1).

**Table 1:** Baseline characteristics

	Overall population (n = 51)	Dose level 1 (n = 10)	Dose level 2 (n = 11)	Dose level 3 (n = 26)	Dose level 4 (n = 4)
**Median age, years (range)**	53 (32-67)	49 (37-64)	49 (32-64)	55 (35-67)	56 (49-56)
**ECOG performance status (%)**					
0	80	80	82	88	25
1	20	20	18	12	75
**Tumour stage (%)**					
1	25	40	18	23	25
2	67	60	82	62	75
3	6	0	0	12	0
4	2	0	0	4	0
**Nodal stage (%)**					
1	10	0	18	12	0
2	53	60	45	54	50
3	37	40	36	35	50
**Involved lymph nodes**					
Median number	9	10	9	7.5	7.5
3-6* (%)	39	20	45	42	50
7-9 (%)	20	30	9	23	0
10-14 (%)	20	10	27	19	25
15-19 (%)	12	20	9	8	25
≥20 (%)	10	20	9	8	0
**Hormone receptor status (%)**					
ER or PgR positive	76	80	64	77	100
ER and PgR negative	24	20	36	23	0
**HER2 status (%)**					
Positive	33	40	27	27	75
Negative	63	60	64	69	25
Unknown	4	0	9	4	0

*One patient had only three involved nodes but was included in all analyses

ECOG, Eastern Cooperative Oncology Group, ER, oestrogen receptor, PgR, progesterone receptor

### Treatment exposure

All but two patients completed all planned cycles of epirubicin and paclitaxel. One patient completed all three cycles of epirubicin but discontinued after only two cycles of paclitaxel because of deterioration of physical condition and breast infection. The second discontinued after the first cycle of epirubicin because of febrile neutropenia. One additional patient was withdrawn before initiation of capecitabine therapy because brain metastases were observed after completion of all planned cycles of epirubicin and paclitaxel.

The mean delivered dose was very close to the planned dose for both epirubicin and paclitaxel. The planned dose of epirubicin or paclitaxel was reduced in 43 of 300 (14.3%) cycles, most frequently because of treatment-related haematological adverse events (23 cycles, 8%). Of the 51 patients recruited (including the three described above who received no study treatment), 72% received all four planned cycles of capecitabine and 14% received three cycles. Four cycles of vinorelbine (day 1 and/or day 8) were given in 67% of the 15 patients accrued to vinorelbine-containing dose levels and 20% received three cycles.

All patients received at least one dose of darbepoetin alfa during the treatment with ET. The majority of patients (71%) received darbepoetin alfa during capecitabine (with or without vinorelbine) therapy: 88% and 58% in the two capecitabine monotherapy cohorts and 90% and 75% in the two vinorelbine-treated cohorts.

### Safety

As defined in the protocol, the safety analysis included all patients who received at least one cycle of chemotherapy. Forty-eight patients received at least one dose of capecitabine (with vinorelbine at dose levels 2 and 4). A total of 176 cycles were administered: 27 to eight patients at dose level 1, 37 to 10 patients at dose level 2, 98 to 26 patients at dose level 3 and 14 to four patients at dose level 4. At dose level 1, one patient experienced grade 4 paraesthesia and grade 3 hand-foot syndrome. At dose level 2 (capecitabine 1000 mg/m^2 ^plus vinorelbine 25 mg/m^2^), five of the 10 patients experienced DLTs (grade 3 hand-foot syndrome in two patients, grade 4 diarrhoea in one patient, febrile neutropenia in two patients [accompanied by grade 4 leucopenia in one of these patients]). The high incidence of DLTs at dose level 2 led to discontinuation of dose level 4, but since there had been only one DLT at dose level 1 (capecitabine monotherapy), accrual continued to dose level 3 (single-agent capecitabine 1250 mg/m^2 ^twice daily). At this dose level, there were four patients with grade 3 hand-foot syndrome, one with grade 4 diarrhoea, and two with febrile neutropenia (Table 2).

**Table 2:** Summary of most common ( > 20%) adverse events and grade 3/4 adverse events in > 5% of patients

	Overall population (n = 51)					Dose level 3 (recommended dose) (n = 26)				
	Grade 1	Grade 2	Grade 3	Grade 4	Total	Grade 1	Grade 2	Grade 3	Grade 4	Total
**Alopecia**	1 (2%)	38 (75%)	0	0	39 (76%)	0	24 (92%)	0	0	24 (92%)
**Nausea**	21 (41%)	9 (18%)	3 (6%)	2 (4%)	35 (69%)	16 (62%)	3 (12%)	1 (4%)	1 (4%)	21 (81%)
**Fatigue**	12 (24%)	17 (33%)	0	0	29 (57%)	8 (31%)	8 (31%)	0	0	16 (62%)
**Stomatitis**	18 (35%)	6 (12%)	2 (4%)	0	26 (51%)	8 (31%)	4 (15%)	2 (8%)	0	14 (54%)
**Hand-foot syndrome**	7 (14%)	6 (12%)	7 (14%)	0	20 (39%)	5 (19%)	4 (15%)	4 (15%)	0	13 (50%)
**Nail disorder**	7 (14%)	11 (22%)	0	1 (2%)	19 (37%)	4 (15%)	6 (23%)	0	0	10 (38%)
**Constipation**	14 (27%)	5 (10%)	0	0	19 (37%)	5 (19%)	3 (12%)	0	0	8 (31%)
**Arthralgia**	6 (12%)	9 (18%)	1 (2%)	0	1 (31%)	4 (15%)	3 (12%)	1 (4%)	0	8 (31%)
**Bone pain**	7 (14%)	5 (10%)	0	0	12 (24%)	5 (19%)	3 (12%)	0	0	8 (31%)
**Paraesthesia**	6 (12%)	4 (8%)	1 (2%)	1 (2%)	12 (24%)	3 (12%)	3 (12%)	0	0	6 (23%)
**Diarrhoea**	5 (10%)	3 (6%)	2 (4%)	2 (4%)	12 (24%)	1 (4%)	2 (8%)	1 (4%)	1 (4%)	5 (19%)
**Headache**	5 (10%)	4 (8%)	2 (4%)	0	11 (22%)	3 (12%)	0	1 (4%)	0	4 (15%)
**Anorexia**	6 (12%)	5 (10%)	0	0	11 (22%)	4 (15%)	1 (4%)	0	0	5 (19%)
**Vomiting**	7 (14%)	2 (4%)	4 (8%)	2 (4%)	15 (29%)	3 (12%)	0	2 (8%)	2 (8%)	7 (27%)
**Febrile neutropenia**	0	0	3 (6%)	3 (6%)	6 (12%)	0	0	2 (8%)	0	2 (8%)
**Neutropenia**	0	1 (2%)	1 (2%)	2 (4%)	4 (8%)	0	1 (4%)	1 (4%)	1 (4%)	3 (12%)
**Impaired healing**	0	0	1 (2%)	1 (2%)	2 (4%)	0	0	1 (4%)	1 (4%)	2 (8%)
**Myalgia**	6 (12%)	2 (4%)	0	0	8 (16%)	6 (23%)	1 (4%)	0	0	7 (27%)
**Hypersensitivity**	3 (6%)	2 (4%)	1 (2%)	0	6 (12%)	3 (12%)	2 (8%)	1 (4%)	0	6 (23%)

The most common adverse events were alopecia, nausea, fatigue, stomatitis and hand-foot syndrome (39%) (Table 2). The majority of adverse events were grade 1 or 2. The most common grade 3/4 adverse events were hand-foot syndrome (14%), vomiting (12%), febrile neutropenia (12%), nausea (10%) and diarrhoea (8%). No grade 3/4 anaemia was observed. Only three patients required red blood cell transfusion; none required thrombocyte transfusion. Median haemoglobin levels were 11.9 g/dL (range 8.4-14.5) at cycle 1 and 13.5 g/dL (range 10.3-14.8) at the end of chemotherapy. Four thrombotic events were reported, two of them in axillary veins related to implanted venous port systems.

The most common reasons for capecitabine or vinorelbine dose reduction or treatment delay were treatment-related non-haematological toxicity in 23 cycles (13%) and haematological toxicity in six cycles (3%). At dose levels 2 and 4 (vinorelbine-containing regimens), 11% and 7% of cycles, respectively, were delayed because of haematological adverse events, compared with 0 and 1% of non-vinorelbine cycles at dose levels 1 and 3. Non-haematological adverse events showed a less clear pattern, leading to dose reduction or delay in 19%, 3%, 13%, and 27% of cycles at dose levels 1, 2, 3 and 4, respectively.

### Efficacy

**Figure 2:**
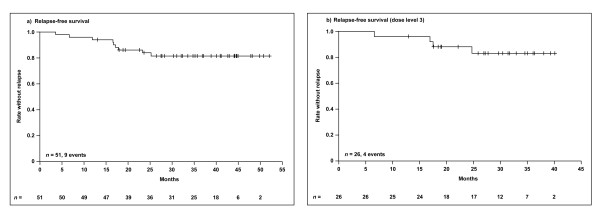
**Kaplan-Meier estimation of relapse-free survival a) overall population; b) dose level 3**.

Median duration of follow-up is 35.2 months. With this follow-up, nine recurrences (18%) and four deaths (8%) have been documented. The estimated 3-year relapse-free survival rate is 82% (95% confidence interval [CI]: 0.71-0.93) (Figure 2), and estimated 3-year overall survival rate is 91% (95% CI: 0.83-1.0).

## Discussion

The findings of this study suggest that capecitabine monotherapy after dose-dense sequential epirubicin and paclitaxel is feasible, whereas the addition of vinorelbine to capecitabine resulted in unacceptable toxicity as predefined by the protocol. Consequently evaluation of the capecitabine-vinorelbine combination after dose-dense epirubicin plus paclitaxel was abandoned in this pilot study.

This is only one of the capecitabine-containing regimens being evaluated in the adjuvant situation. In a randomised, neoadjuvant trial, the combination of docetaxel and capecitabine demonstrated a significantly higher pathologic complete response rate than doxorubicin plus cyclophosphamide [14] and early results from the randomised European Cooperative Trial in Operable Breast Cancer II (ECTOII) trial demonstrated more favourable efficacy with capecitabine-containing regimens than those without capecitabine [15]. In contrast, in the GeparQuattro study, von Minckwitz et al. were unable to show additional benefit when capecitabine was added to a highly effective neoadjuvant taxane- and anthracycline-containing regimen [16].

In the adjuvant setting, results of a randomised, phase III trial demonstrated favourable efficacy when capecitabine was given in combination with docetaxel followed by epirubicin, cyclophosphamide and capecitabine (TX→CEX) versus docetaxel alone followed by 5-fluorouracil (5-FU), epirubicin and cyclophosphamide (T→CEF). The estimated 34% risk reduction in relapse-free survival appears substantial [17]. In contrast, a study comparing adjuvant capecitabine monotherapy to either AC or CMF combination therapy in older patients showed the latter regimens to be superior [7]. These results allow no definitive conclusion about the role of capecitabine in the adjuvant setting.

Currently, the impact of adding vinorelbine to capecitabine after anthracycline and taxane exposure is not clear. In the absence of randomised data in EBC, extrapolation from the metastatic setting possibly provides the best indicator. The combination of both drugs is well tolerated and has shown substantial efficacy in MBC. However, cross-trial comparison does not clearly indicate that addition of vinorelbine to capecitabine increases the efficacy in patients previously exposed to anthracyclines and taxanes [11,18,19]. Therefore, without randomised data in either the metastatic or adjuvant setting, no firm conclusions can be drawn.

In the neoadjuvant GEPARTRIO trial, patients who did not show a response after two cycles of docetaxel, doxorubicin and cyclophosphamide (TAC) were randomised to either four further cycles of TAC or four cycles of capecitabine and vinorelbine. In contrast to our findings, administration of vinorelbine and capecitabine after TAC was feasible, resulting in similar efficacy but better tolerability than continuing TAC [20]. However, to our knowledge no other trials have tested the combination of capecitabine-vinorelbine as front-line therapy in patients with EBC, and TAC was not dose dense and escalated as in our study.

Compared with the AGO trial evaluating the ddETC regimen, the capecitabine-containing regimens were well tolerated. At both capecitabine doses, gastrointestinal toxicities and hand-foot syndrome were among the most frequently reported grade 3/4 non-haematological adverse effects. However, there were fewer cyclophosphamide-related toxicities, such as stomatitis, pain, infection and skin toxicity. As expected, grade 3 hand-foot syndrome and grade 4 diarrhoea were dose limiting, occurring in four patients and one patient, respectively, treated at the higher dose of 1250 mg/m^2 ^twice daily. Since this trial was initiated, a randomised, phase III trial of first-line capecitabine monotherapy versus classical CMF for MBC demonstrated that a dose of 1000 mg/m^2 ^twice daily is very active, resulting in a significant survival benefit [5]. Moreover, retrospective analyses and non-randomised trials in MBC suggest that this lower dose is associated with better tolerability without compromising efficacy [21-23]. As a consequence the lower dose is frequently used in clinical practice. The evaluation of lower dose levels was not planned because when the present study was designed, it was assumed that dose levels 1 and 2 were feasible and well tolerated in the metastatic situation, a hypothesis confirmed by studies published after initiation of our study [10-12].

For a population with a median of nine positive axillary lymph nodes, the 82% 3-year relapse-free survival rate after 35.2 months' median follow-up is within the range reported for dose-dense anthracycline-taxane-cyclophosphamide regimens [2,3,24,25]. Long-term follow-up is ongoing.

Darbepoetin alfa was effective in preventing grade 3/4 anaemia. However, since starting this phase I/II study, the general consensus on the role of epoetin-containing drugs in cancer patients has changed substantially and current guidelines demand a more cautious use of these compounds as in our study [26,27]. This is based on some data suggesting poorer overall survival in non-anaemic patients receiving epoetin compared with placebo [28] and increased risk of thromboembolism [29]. In the 2007 update of the St. Gallen guidelines for management of EBC [30] haematopoietic growth factors were considered by most panel members to have a role in patients with a clinical indication, although only a minority supported their routine use. The current St. Gallen statement of 2009 does not mention this topic. However, analyses from the ddETC trial indicated that epoetin administration enabled maintenance of a stable median haemoglobin value and avoided the need for blood transfusions, without any negative impact on relapse-free and overall survival [3].

## Conclusion

Taken together, our results show that after intensified dose-dense epirubicin and paclitaxel, the combination of capecitabine and vinorelbine appears unfeasible. These results influenced the design of the recently completed GAIN (German Adjuvant Intergroup Node-positive) trial. Consequently, patients in GAIN were randomised to either ddETC or an investigational epirubicin and cyclophosphamide followed by capecitabine and paclitaxel (EC→XP) arm (four 14-day cycles of epirubicin 112.5 mg/m^2 ^plus cyclophosphamide 600 mg/m^2^, followed by four 21-day cycles of capecitabine 1000 mg/m^2 ^twice daily administered on days 1-14 in combination with weekly paclitaxel 67.5 mg/m^2 ^for 10 weeks). The low relapse rate observed in the study described here supports the concept of dose-dense therapy. In this context, our results should help to optimise the design of further clinical trials in high-risk breast cancer.

## Competing interests

VMü has received speaker honoraria from Amgen, Sanofi-Aventis, Pierre-Fabre and Roche and research funding from Sanofi-Aventis and Roche. CT has received speaker honoraria from Amgen, Sanofi-Aventis, Pierre-Fabre and Roche and research funding from Sanofi-Aventis and Roche. MS has no conflict of interest to declare. CJ has received speaker honoraria from Amgen, Sanofi-Aventis, Pierre-Fabre and Roche and research funding from Roche. VH has received speaker honoraria from Astra Zeneca Oncology and Novartis. AL has received speaker honoraria by Bristol Myers Squibb and Roche. HB has no conflict of interest to declare. VMö has received speaker honoraria from Amgen, Bristol Myers Squibb, GlaxoSmithKline, Novartis, Pfizer and Roche and research funding from Amgen, BMS, Roche and Johnson and Johnson. AH and MG have no competing interests to declare.

## Authors' contributions

VMü contributed to patient recruitment and was responsible for writing of the manuscript; AL contributed to patient recruitment; MS contributed to patient recruitment; MG contributed to patient recruitment; CJ contributed to patient recruitment; VH contributed to patient recruitment; AH performed statistical analyses; CT contributed to patient recruitment, manuscript writing and study design; HB contributed to patient recruitment; VMö contributed to patient recruitment, study design and writing of the manuscript. All authors read and approved the final manuscript.

## Pre-publication history

The pre-publication history for this paper can be accessed here:

http://www.biomedcentral.com/1471-2407/10/430/prepub
